# NAVIGATIONAL BARRIERS TO ADVANCE DIRECTIVE COMPLETION AMONG OLDER LATINOS WITH CHRONIC ILLNESS

**DOI:** 10.1093/geroni/igz038.644

**Published:** 2019-11-08

**Authors:** Frances R Nedjat-Haiem

**Affiliations:** San Diego State University, School of Social Work, San Diego, California, United States

## Abstract

This study examined navigational barriers to advance care planning (ACP) and advance directive (AD) completion among older Latinos with chronic illness. Older Latinos over the age of 50 were randomized to education only versus education plus counseling (N=61; education only n=31, education/counseling n=30). Exploratory factor analyses and logistic regression show relationships between navigational barriers, depression, anxiety and AD documentation. Barriers to ACP included difficulty understanding the doctor, fears about hearing bad news, worries about illness getting worse, and difficulties due to language, family conflict, and limited family discussions about dying. Treatment group was most significant contributing factor to AD completion (OR=4.213, CI=1.126 – 15.760, p=<.01). Findings indicate the importance of examining a variety of factors that could influence AD documentation. This study contributes to the literature by identifying the need for targeted and culturally relevant interventions that promote ACP education, counseling, communication, support, and AD documentation among older Latinos.

